# Effects of ranibizumab (Lucentis®) and bevacizumab (Avastin®) on human corneal endothelial cells

**DOI:** 10.1186/s12886-018-0978-9

**Published:** 2018-12-11

**Authors:** Patrick R. Merz, Nina Röckel, Seda Ballikaya, Gerd U. Auffarth, Ingo Schmack

**Affiliations:** 1 Department of Ophthalmology, University of Heidelberg, Lions Eye Bank, Im Neuenheimer Feld 400, 69120 Heidelberg, Germany; 2 Department of Ophthalmology, Goethe-University Frankfurt, Theodor-Stern-Kai 7, 60590 Frankfurt/Main, Germany

**Keywords:** Corneal neovascularization, Corneal endothelial cells, Corneal angiogenesis, Vascular endothelial growth factor, Ranibizumab, Bevacizumab

## Abstract

**Background:**

Ingrowth of newly formed blood and lymph vessels (angiogenesis) from the limbus region into the cornea can be treated successfully by subconjunctival application of antiangiogenic agents. Currently, there are several angiogenesis inhibitors from various manufacturers available, such as vascular endothelial growth factor (VEGF) antibodies. The aim of the study was to investigate potential cytotoxic effects of two anti-VEGF agents, ranibizumab (Lucentis®) and bevacizumab (Avastin®) on the human corneal endothelium.

**Methods:**

Human donor corneas, not suitable for corneal transplantation, were organ-cultured in the presence of either ranibizumab (Lucentis®) or bevacizumab (Avastin®) at different concentrations (group 1: 250 μg / ml, group 2: 25 μg / ml, group 3: 2.5 μg / ml) for a period of up to 4 weeks. Microscopic imaging for endothelial cell counting, detection of morphologic alterations of the endothelium, and molecular biology testing (Enzyme-linked Immunosorbent Assay [ELISA]) for metabolic changes was performed.

**Results:**

Background-corrected results showed neither a significant lactate dehydrogenase (LDH) change with increasing culturing time nor a significant difference between ranibizumab (Lucentis®) and bevacizumab (Avastin®) treatment. The endothelial cell density revealed also no statistically significant difference between the two treatment groups with ranibizumab (Lucentis®) and bevacizumab (Avastin®) at all concentrations tested in this study.

**Conclusions:**

In this study, the anti-angiogenic agents ranibizumab (Lucentis®) and bevacizumab (Avastin®) demonstrated no cytotoxic effects on the corneal endothelium of human organ-cultured donor corneas over the limited study time period of 4 weeks. However, based on the study design (in-vitro) and the limited follow-up period, no conclusions on potential long-term effects can be drawn.

## Background

Antiangiogenic substances have been studied extensively for their potential cytotoxic effects on human corneal cells [1–4]. The majority of cell culture and animal studies did not show any cytotoxic changes of the corneal endothelium in presence of anti-angiogenic agents. However, single studies suggest that expression of transmembrane proteins and Na+/K+ -ATPase may occur under the influence of antiangiogenic agents, possibly leading to adverse effects on corneal stroma homeostasis [5–7].

Corneal avascularity and transparency are basic prerequisites for the high imaging quality of the human eye. They are based essentially on the ultrastructure of the corneal stroma and a close interplay of angiogenic and antiangiogenic substances. In connection with inflammation, mechanical or chemical trauma to the cornea; however, this balance may be disturbed with consecutive ingrowth of blood and lymph vessels (angiogenesis) from the limbal region [8, 9]. These changes are usually associated with decreased corneal transparency and reduction of visual acuity [10]. Furthermore, corneal neovascularization and the associated immunological reactions contribute to an increased risk of failure and rejection after corneal transplantation [11–13]. Tang and co-workers showed that the endothelial growth factor VEGF (vascular endothelial growth factor) with its isoforms and surface receptors is crucial for the development of corneal neovascularization [14]. VEGF is derived from a variety of corneal cells, i.e. epithelia, keratocytes and endothelial cells [15].

Thanks to advances in angiogenesis research in recent years, specific angiogenesis inhibitors are now available for topical application [16]. They include VEGF antibodies such as ranibizumab (Lucentis®) and bevacizumab (Avastin®) as well as anti-angiogenic antisense oligonucleotides.

The long-term goal is to develop a specific dose-response curve related to subconjunctival and intracameral administration of ranibizumab and bevacizumab for patients at increased risk of rejection after corneal transplantation. The purpose of this study was to evaluate ranibizumab (Lucentis®) and bevacizumab (Avastin®) for potential cytotoxic effects on the corneal endothelium of human in organ-cultured donor corneas.

## Methods

### Organ cultivation of human donor corneas

All corneas used in this study were taken under the legal responsibility of the Department of Ophthalmology, Lions Eye Bank (Federal authority Paul-Ehrlich-Institut: PEI.G.11601.01.1) within the facilities of the University Hospital Heidelberg. Forty-six human donor corneas were included in the study. They were not suitable for transplantation for one or more of the following reasons:

- Endothelial Cell Count < 2000 Cells/mm^2^
- Positive Conjunctival Swab
- Positive or Unclear Serology
- Contamination of Fellow Eye

Individual exclusion criteria for each donor cornea are summarized in Table 1.

**Table 1:** Demographic characteristics of the study samples

	ranibizumab (Lucentis®)	bevacizumab (Avastin®)
Sex (No. of patient)		
Female	9 (37.5%)	11 (50.0%)
Male	15 (62.5%)	11 (50.0%)
Total	24	22
Age, years (No. of patients)		
50–59	3 (12.5%)	2 (9.1%)
60–69	4 (16.7%)	7 (31.8%)
70–79	2 (8.3%)	6 (27.3%)
80+	15 (62.5%)	7 (31.8%)
Mean [Min;Max]	78.7 [51.0;93.8]	73.8 [58.7;88.8]
Causes of death (No. of patients)		
Cancer	5 (20.8%)	5 (22.7%)
Cerebrovascular disease	7 (29.2%)	4 (18.2%)
Coronary heart disease	8 (33.3%)	11 (50.0%)
Respiratory diseases	4 (16.7%)	2 (9.1%)
Culture conditions		
culture time [d] (Mean [Range])	23.0 [11.0;42.0]	28.0 [10.0;43.0]
death-to-enucleation-time [h] (Mean [Range])	26.7 [6.3;49.4]	29.6 [10.9;54.4]
death-to-culture-time [h] (Mean [Range])	52.2 [10.2;96.9]	61.7 [20.0;138.5]
Reasons for Unsuitability of Donor Corneas		
Endothelial Cell Count < 2000 Cells/mm^2^	4 (16.7%)	5 (22.7%)
Positive Conjunctival Swab	6 (25.0%)	11 (50.0%)
Positive or Unclear Serology	12 (50.0%)	5 (22.7%)
Contamination of Fellow Eye	2 (8.3%)	1 (4.6%)

In the case of poor quality of the corneas (endothelial cell count < 500 cells per mm^2^, endothelial cell necrosis > 50%) or contamination of the culture medium, the respective test part was stopped and repeated with a new cornea.

Written consent of relatives to use the tissue for scientific purposes was present for all corneas used in this study.

Corneas included in this study were randomly assigned to one of 6 study groups or the control group and cultured for 1, 3, 7 or 14 days. Three corneas were used per time point in each group. The corneas were cut in half (2 corneas were quartered). One half was used in the control group and the other half in one of the three study groups. All corneal segments were cultivated in 48-well plates containing 2 ml of respective liquid (BSS, medium and agent if any). Study groups contained different concentrations of ranibizumab (Lucentis®) or bevacizumab (Avastin®) (group 1: 250 μg / ml, group 2: 25 μg / ml, group 3: 2.5 μg / ml). Study agents were diluted in balanced salt solution (BSS). One milliliter of each dilution was added to 1 ml culture medium (Cornea Max®, Eurobio). In contrast, corneas of the control group were cultured in 1 ml culture medium supplemented with 1 ml of undiluted BSS solution, only.

### Determination of endothelial cell count and assessment of cell morphology

Corneal endothelial cells were qualitatively and quantitatively evaluated for abnormalities at baseline using a specular microscope (EM-3000, Tomey Corporation, Aichi, Japan). Only corneas with a coefficient of variation (CV: defined as standard deviation of the cell size expressed as percentage divided by the mean cell size) below 40 indicating low polymegathism levels and a hexagonality over 50% were used for culturing. Before and after 1, 3, 7 or 14 days of cultivation all corneas were stained with 0.06% trypan blue (VisionBlue, D.O.R.C., Zuidland, The Netherlands) for evaluation of endothelial cell viability. In detail, two drops of 0.06% sterile isotonic trypan blue solution were applied to the corneal specimen’s endothelial cells facing up. After incubation for 60 s, corneas were rinsed with balanced salt solution. Subsequently endothelial cell counting was performed manually by light microscopy (Olympus CKX41, Olympus Europa Se & Co Kg, Hamburg, Germany). We performed cell counting in real time with the support of the NAVIS software (NIDEK Technologies Srl, Padova, Italy). Endothelial cells were counted within a central rectangle in L-configuration. The endothelial cell count of the vivid cells was calculated as endothelial cells/mm^2^ (ECD).

In addition, morphologic parameters such as endothelial cell morphology (cell size and shape) and cell viability were evaluated at each time point. All corneas were dehydrated for 24 h in a dextran-containing culture medium (Cornea Jet®, Eurobio) before cell counting.

### Cytotoxicity assay

Endothelial cells were evaluated for potential cell damage by determination of LDH activity within the culture medium. LDH is secreted by damaged endothelial cells. The enzyme is capable of converting the pale yellow test reagent into red (*tetrazolium*) which is colorimetrically detectable by a photometer at a wavelength of 490 nm. The measured light intensity is directly proportional to the amount of LDH released into the supernatant of the culture medium [17]. In detail, 96-well microtiter culture plates were sensitized by incubating with 100 μLVEGF at a concentration of 0.15 mg/L in coating buffer (1 mol/L carbonate–bicarbonate buffer, pH 9.6) overnight at + 4 °C. Plates were washed 4-times with PBS containing 0.05% Tween 20. The remaining unspecific protein-binding sites were blocked by incubation with 200 μL blocking buffer (PBS containing 1% BSA) for 2 h at room temperature. Plates were subsequently washed as follows: After adding 100 μL of 1:100 diluted standards, quality controls (QCs), or test samples plates were incubated for 1 h at 37 °C in an iEMS incubator-shaker (Labsystems, Helsinki, Finland) with a subsequent rinsing. Then, 100 μL of conjugated-anti IgG antibodies diluted in 1% PBS-BSA was added to each well for 1 h at room temperature and subsequently rinsed. Finally, 100 μL o-Phenylenediamine (OPD) (prepared by dissolving tablet sets in 20 mL distilled water) was added and the reaction was allowed to develop at room temperature in the dark. The color reaction was stopped by adding 50 μL of 2 mol/L sulfuric acid to each well. Reading was performed at two wavelengths (492 and 620 nm) using an iEMS ELISA plate reader (Labsystems). The plate background absorbance at 620 nm was subtracted from the absorbance at 492 nm.

## Results

### Determination of endothelial cell count and assessment of cell morphology

Corneas showed variable endothelial cell findings in the study and control groups. Endothelial cell counts did not shown any statistical significant differences after treatment with ranibizumab (Lucentis®) and bevacizumab (Avastin®). The overall cell loss and the corneal area were comparable within study groups (Table 2).

**Table 2:** Clinical Characteristics of the Study Samples

	ranibizumab (Lucentis®)	bevacizumab (Avastin®)
Endothelial Cell Density ECD [Cells/mm^2^]		
Before Treatment (Mean [Range])	2441 [1700;3200]	2234 [1700;2800]
After Treatment 0 μg/ml (control)	1847	1644
After Treatment 2.5 μg/ml	1511	1587
After Treatment 25 μg/ml	1460	1417
After Treatment 250 μg/ml	1715	1352
Cell loss 0 μg/ml (control)	18.1%	15.8%
Cell loss 2.5 μg/ml	22.5%	18.0%
Cell loss 25 μg/ml	25.7%	25.8%
Cell loss 250 μg/ml	24.2%	32.5%
Corneal Area [mm^2^] (Mean)	75.0	67.0

**Fig. 1:**
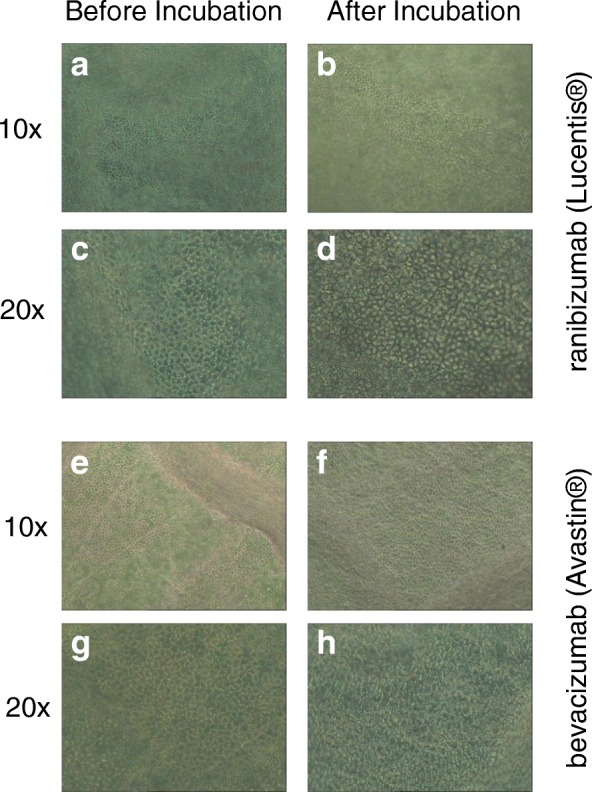
Hexagonal shaped single layer human endothelial corneal cells of donor corneas before (**a**, **c**, **e**, **g**) and after (**b**, **d**, **f**, **h**) incubation with 250 μg/ml ranibizumab (Lucentis®) or bevacizumab (Avastin®) at indicated magnifications. No statistical significant difference between before and after could be detected

**Fig. 2:**
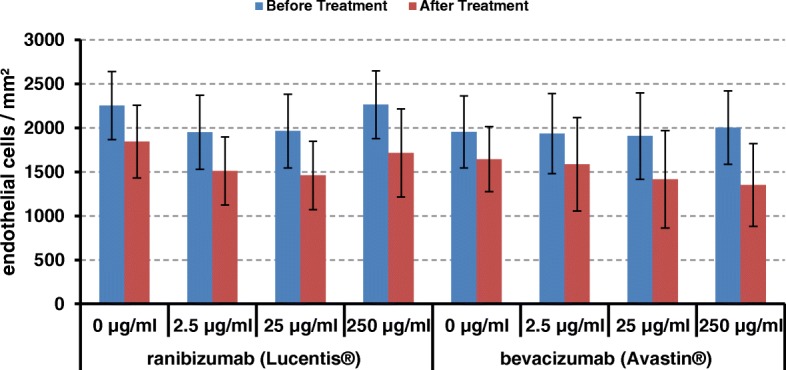
Endothelial cell density (mm^2^) before (blue) and after (red) incubation with ranibizumab (Lucentis®) or bevacizumab (Avastin®)

Figure 1 illustrates hexagonal shaped human endothelial corneal cells at a magnification of 10× and 20× obtained by light microscopy. No major differences in regard to cell morphology (size, shape) and viability were detected among the study groups and the controls over time (Fig. 1). No statistical significant endothelial cell loss occurred during incubation with ranibizumab (Lucentis®) and bevacizumab (Avastin®) compared to the control group. A significant natural loss of cells as usually seen during prolonged incubation in vitro is illustrated in the control groups (Fig. 2).

#### Cytotoxicity assay

**Fig. 3:**
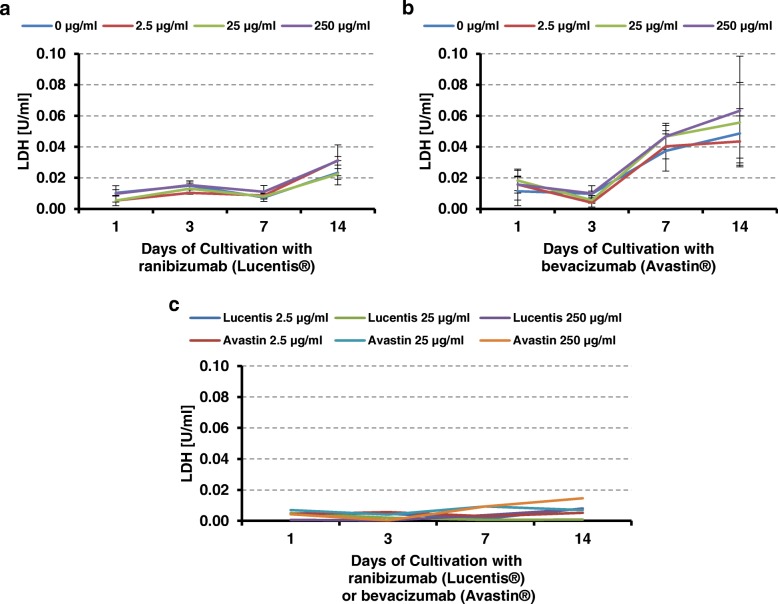
Overview of measurements of LDH values as a function of culture time **a**: LDH values for 0–250 μg / ml ranibizumab (Lucentis®) **b**: LDH values for 0–250 μg/ml bevacizumab (Avastin®); **c**: LDH values for ranibizumab (Lucentis®) or bevacizumab (Avastin®) after background correction with 0 μg/ml control group

The cytotoxic effect on the endothelial cells was evaluated by photometric determination of the lactate dehydrogenase (LDH) enzyme activity in the culture medium. Under physiologic conditions, the total LDH concentration in human sera ranges between 0.125 and 0.25 μg / ml. Background-corrected results (Fig. 3 a-c) show no significant LDH changes in regard to the cultivation time as well as any statistically significant differences among the study groups (ranibizumab (Lucentis®) versus bevacizumab (Avastin®)) There is only a slight trend independent from the antiangiogenic concentration that for bevacizumab (Avastin®) after 7 days and for ranibizumab (Lucentis®) after 3 days an increase in the LDH concentration could be observed.

## Discussion

The innermost cell layer of the cornea, the corneal endothelium, plays a key role in maintaining corneal transparency. As a metabolically active cell assemblage, they are involved in a continuous outflow of extracellular fluid from the corneal stroma into the anterior chamber, thus counteracting the constant osmotic gradient of the protein component of the corneal stroma [18–20].

Preserving of the morphology and functionality of the corneal endothelium as well as dehydration of the corneal stroma can be permanently impaired by an altered expression behavior of certain key genes. Inhibition of the underlying signaling cascade by adequate and early topical or systemic anti-VEGF therapy plays an important role in inhibiting corneal angiogenesis and lymph angiogenesis [21, 22]. The inhibitory effects of bevacizumab on corneal angiogenesis have been demonstrated previously in various laboratory and animal models. In addition, an antiangiogenic effect of bevacizumab could be demonstrated in the context of different anterior eye segment disorders after topical application, subconjunctival and intracameral administration [22–26].

Experimental studies showed similar inhibitory effects of topical (eye drops) and subconjunctival administrated ranibizumab (Lucentis®) [27]. In addition, a non-toxic effect to the corneal endothelium in patients treated repeatedly with intravitreal injections of bevacizumab or ranibizumab due to age-related macular degeneration (AMD) is already known [4, 7]. The good tolerability of both antiangiogenic substances could be confirmed in our in vitro laboratory study on human corneal endothelial cells. Since the effects of ranibizumab and bevacizumab on human corneal endothelial cells have currently only been investigated secondary to intravitreal injections inpatients with age-related AMD and in cell culture models it is of particular interest to analyze the potential influence of both substances on the corneal endothelium after topical (eye drop), subconjunctival and intracameral application [7, 28] .

In addition, the safety and toxicity of ranibizumab and bevacizumab has only performed in endothelial cell culture or animal models. Studies on human donor corneas are lacking. Best of our knowledge, the present study was the first laboratory investigation demonstrating that both anti-angiogenic substances – ranibizumab and bevacizumab – did not show cytotoxic effects on the human endothelium in corneal donor tissue. Cell culture models using human retinal pigment epithelium (ARPE-19) cells were able to confirm out findings with no toxic effect after single or repeated application of various doses of VEGF antagonists under normal and oxidative stress [29]. In addition, Aflibercept did not show any negative effects on retinal cell lines at all [30]. Also bevacizumab might potentially affect mitochondria functionality differently from ranibizumab and aflibercept, ranibizumab and aflibercept at clinical doses protect against cytotoxicity in human ARPE-19 cells [31]. It could even been excluded that normal doses of ranibizumab and aflibercept produce mitochondrial toxicity or cell death. However, bevacizumab and ziv-aflibercept are proven to have mild mitochondrial toxicity at clinically relevant doses in ARPE-19 cells [32]. Additionally studies indicate that anti-VEGF agents can interfere with the physiological functions of RPE cells under high-glucose conditions leading to decrease in cell viability and proliferation [33].

There is moderate evidence that ranibizumab and bevacizumab are equivalent in terms of efficacy and toxicity [34]. Additionally, ranibizumab and aflibercept showed a similar endophthalmitis rates after intravitreal application compared to bevacizumab [35]. In general, intraocular application of low doses of VEGF does not induce irreversible toxic retinal damage [36]. Furthermore it was stated that aflibercept and ranibizumab are more effective than bevacizumab against type 1 choroidal neovascularization because of the lower amount of VEGF in the posterior chamber using aflibercept and ranibizumab compared to bevacizumab [37].

No differences in overall safety between the three antiangiogenic drugs that are currently available but results are imprecise for cardiovascular events and death [38]. Therefore, storage of antiangiogenics within cells of intraocular tissues as observed in vivo after intravitreal injection into monkey eyes, may result in unknown side effects during long-term treatment of patients. Only the antibody bevacizumab (not the antibody fragment ranibizumab) was shown to accumulate in both retinal endothelial cells and pigment epithelial cells during prolonged treatment. This might be of importance because patients are often treated for several years and so additional long-term side effects may be realized in the future [39, 40].

Therefore, key gene expression assays should contribute to a better understanding of possible long-term effects of anti-VEGF therapy on the functional properties of corneal endothelial cells. It may also be possible to reduce the risk of rejection following corneal transplantation in the future by supplementing gene therapy. The identification of new key genes would make a corresponding contribution to this. The results of our study showed that there were no statistically significant cytotoxic effects of ranibizumab and bevacizumab on the human endothelial cells at various concentrations.

## Conclusions

In this short time study, no cytotoxic effects of either ranibizumab (Lucentis®) or bevacizumab (Avastin®) on the corneal endothelium of human organ-cultured donor corneas were observed. After deducting the control group, neither a significant increase in LDH expression nor a significant difference between ranibizumab (Lucentis®) and bevacizumab (Avastin®) incubation could be detected. Within the 4 weeks of incubation there was no statistically significant higher endothelial cell loss during incubation with ranibizumab (Lucentis®) and bevacizumab (Avastin®). There were no obvious morphological differences between the two study groups over time and compared to their respective controls indicating no difference in cytotoxicity between ranibizumab (Lucentis®) and bevacizumab (Avastin®).

As a serious limitation of this study it has to be emphasized that all conclusions are based on a study time of only 4 weeks. Negative effects occurring after prolonged incubation time can therefore not be excluded.

## Abbreviations

AMD: Age-related macular degeneration
BSA: Bovine serum albumin
BSS: Balanced salt solution
ELISA: Enzyme-linked immunosorbent assay
LDH: Lactate dehydrogenase
OPD: Ortho-phenylenediamine
PBS: Phosphate buffered solution
VEGF: Vascular endothelial growth factor
